# Molecular targets for anti-oxidative protection of green tea polyphenols against myocardial ischemic injury

**DOI:** 10.7603/s40681-014-0023-0

**Published:** 2014-11-20

**Authors:** Shih-Rong Hsieh, Wei-Chen Cheng, Yi-Min Su, Chun-Hwei Chiu, Ying-Ming Liou

**Affiliations:** 1 Department of Life Sciences, National Chung-Hsing University, 402 No. 250, Kuokang Road, Taichung, Taiwan; 2 Department of Cardiovascular Surgery, Taichung Veterans General Hospital, 407 Taichung, Taiwan; 3 Institute of Bioinformatics and Structural Biology, National Tsing Hua University, 300 Hsinchu, Taiwan; 4 Rong Hsing Research Center for Translational Medicine, National Chung Hsing University, 402 Taichung, Taiwan

**Keywords:** Cardio-protection, Green tea polyphenols (GTPs), Ischemic heart disease, Oxidative stress, Proteomics

## Abstract

Ischemic heart disease is the leading cause of death worldwide. An improved understanding of the mechanisms involved in myocardial injury would allow intervention downstream in the pathway where certain drugs including natural products could be efficiently applied to target the end effectors of the cell death pathway. Green tea polyphenols (GTPs) have potent anti-oxidative capabilities, which may account for their beneficial effects in preventing oxidative stress associated with ischemia injury. Although studies have provided convincing evidence to support the protective effects of GTPs in cardiovascular system, the potential end effectors that mediate cardiac protection are only beginning to be addressed. Proteomics analyses widely used to identify the protein targets for many cardiovascular diseases have advanced the discovery of the signaling mechanism for GTPs-mediated cardio-protection. This review focuses on putative triggers, mediators, and end effectors for the GTPs-mediated cardio-protection signaling pathways engaged in myocardial ischemia crisis, allowing a promising natural product to be used for ameliorating oxidative stress associated with ischemic heart diseases.

## 1. Introduction

Green tea polyphenols (GTPs) have attracted much interest in prevention of atherosclerosis and cardiovascular diseases [1-7]. Epidemiological studies have established a close correlation between the consumption of green tea and protection against cardiovascular diseases and risk factors [8-12]. Other experimental studies on myocardial ischemia injury have also suggested that the cardio-protective effect of GTPs is associated with the scavenging of active-oxygen radicals, the modulation of redox- sensitive transcription factors (e.g., NFκB, AP-1), the reduction of STAT-1 activation and Fas receptor expression, an increase in NO production, the exertion of positive inotropic effects, and the modulation of myofilament Ca^2+^ sensitivity [13-21]. However, limited information is known for the potential end effectors in the GTPs-conducted signaling pathways for cardiac protection. This review intends to increase our understanding on the GTPs-mediated cardio-protective mechanism by which molecular targeting for their anti-oxidative interventions on myocardial ischemic disorder is discussed.

## 2. Anti-oxidative capacities of GTPs

Oxidative stress describing an imbalance between the generation and clearance of reactive oxygen species (ROS) in cells has been associated with hypoxia or myocardial ischemia, and likely contributes to the progression of cardiovascular diseases [22]. Accumulating evidence also indicates that redox-sensitive signaling pathways *via* the effects of generation of ROS or reactive nitrogen species (RNS) or reactive lipid derived aldehydes (LDAs) are essentially involved in the pathological stress of heart cells [23]. Accordingly, molecular targeting for anti-oxidative interventions on redox signaling pathways may provide a therapeutic approach to ameliorate the risk and progression for heart diseases.

GTPs have potent antioxidant and radical-scavenging properties, which may partially account for their cardio-protective effects [24]. *In vitro*, they have been shown to scavenge ROS or RNS, chelate metal ions, prevent the activation of redox-sensitive transcription factors, inhibit ROS generating enzymes, and increase antioxidant enzymes [25, 26]. The major catechins in GTPs include epicatechin (EC), epigallocatechin (EGC), epicatechin- 3-gallate (ECG), and epigallocatechin-3-gallate (EGCG) [27, 28]. These compounds (i.e. biologically active polyphenolic flavonoids) contain two or more aromatic rings, each bearing at least one aromatic hydroxyl connected with a carbon bridge. EGCG is the most physiologically potent compound, and primarily accounts for the biological effects of green tea [2]. Studies with a cell line of H9c2 rat cardiomyoblasts associated with H_2_O_2_- induced oxidative stress also demonstrated the protective role of EGCG against oxidative injury and cell death caused by ROS and cytosolic Ca^2+^ overload in cardiac cells [29-31].

**Fig. 1:**
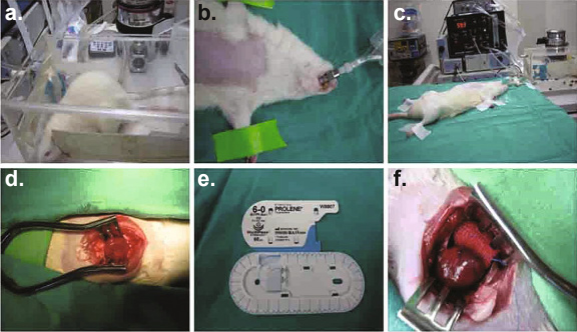

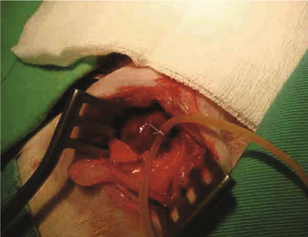
Myocardial ischemia models of chronic myocardial infarction (MI) and transient ischemia-reperfusion (IR) created in rats by ligating the left anterior descending coronary (LAD).

MI surgical model

IR surgical model: 20 min ischemia (I) followed by reperfusion (R)

## 3. GTPs mediated cardiac protection against myocardial ischemic injury

### 3.1. Animal models for cardiac adaptation to oxidative stress and myocardial ischemia

Two different myocardial ischemia models (Figure 1) associated with chronic myocardial infarction (MI) and transient ischemiareperfusion (IR) were created in rats by ligating the left anterior descending coronary (LAD) for studying myocardial ischemic injury [13, 14]. In the MI model, severe myocardial infarction was found in post-MI rats [14], while the IR model involving brief regional ischemia for 20 min followed by subsequent reperfusion showed no severe infarcted injury [13]. These findings suggested that brief regional ischemia followed reperfusion may lead to activate pathways that either preserve cell viability (preconditioning) or lead to cell death (IR injury). In contrast, irreversible MI caused by death of myocytes, presumably as a result of both necrosis and apoptosis, mostly appears within the infarct and periinfarct regions [32-35].

Reperfusion injury of ischemic tissue is known to be accompanied by the production of ROS and Ca^2+^ overload in injured cardiomyocytes [36-43]. The rise in cytosolic Ca^2+^ levels could induce mitochondrial Ca^2+^ ([Ca^2+^]m) accumulation *via* the mitochondrial Ca^2+^ uniporter and the increased ROS production. Both the [Ca^2+^]m overload and increased ROS generation would induce opening of the mitochondrial permeability transition pore (mPTP) and rupture of the plasma membrane, triggering cell death [44, 45].

### 3.2. Pretreatment of GTPs protects myocardial ischemia injury in post-IR rats

A previous study by Miwa *et al*. using isolated hearts perfused with a Langendorff’s apparatus showed that GTPs pre-treatment (1 mM, 35 ml/day for 14 days), administered orally prior to surgery, could protect hearts from oxidative stress after reperfusion and avoid cell edema [46]. This result suggested that GTPs might be used as a novel method for preparative cardiac surgery in the future [46]. In addition, other study using a post-IR model in rats also demonstrated that GTPs pretreatment for 4 hours prior to IR injury protects cardiomyocytes by preventing cytosolic Ca^2+^ overload, myofibril disruption, and alterations in adherens and gap junction protein expression and distribution [13].

### 3.3. GTPs attenuate myocardial remodeling injury in post-MI rats

**Fig. 2:**
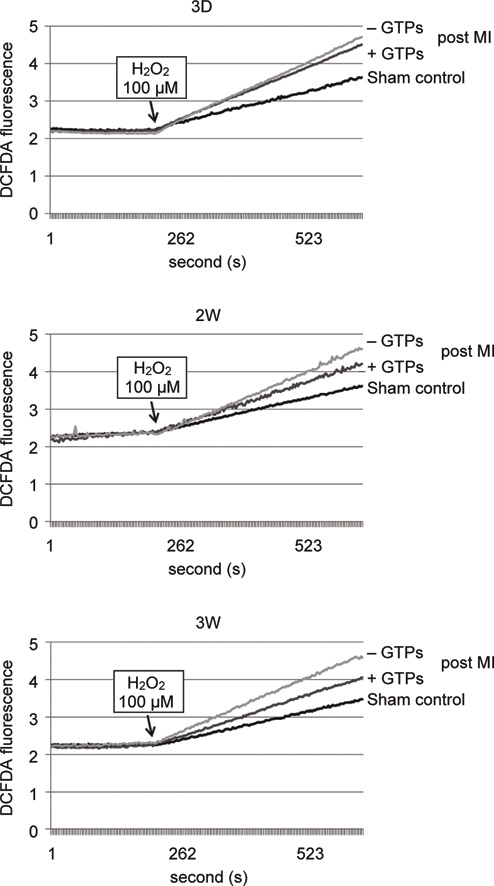
The rate of intracellular ROS produced in cardiomyocytes extracted from post MI rats with or without GTPs treatment for 3 days (3D), 2 weeks (2W), and 3 weeks (3W). Intracellular ROS formation was measured by the fluorescence changes of 2’,7’-dichlorofluorescin diacetate (DCF-DA) in cardiomyocytes with fluorescence spectrophotometry. The fluorescence excitation maximum for DCF-DA was 495 nm, and the corresponding emission maximum was 527 nm.

**Fig. 3:**
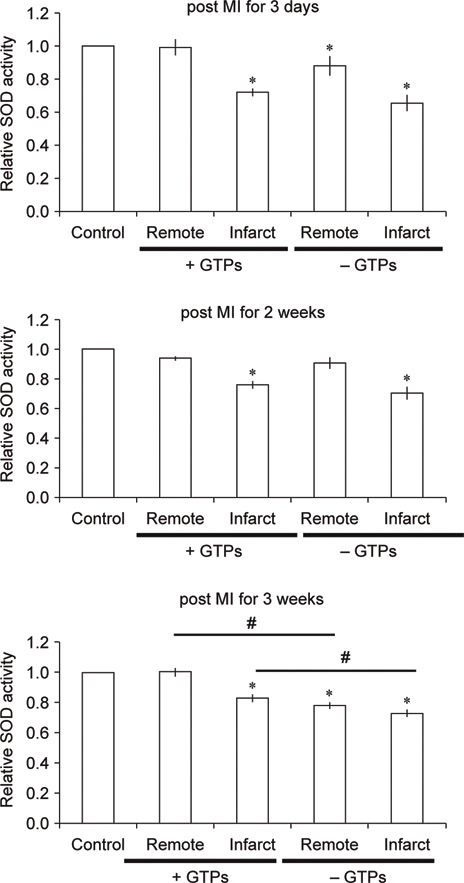
The relative activity of SOD in cardiac tissues of sham control, post MI rats with or without GTPs treatment for 3 days, 2 weeks, and 3 weeks.

Myocardial infarction (MI) largely resulting from cardiac ischemic injury often undergoes to cardiac remodeling process, which may cause secondary damage to the heart tissue by excessive ROS and free radicals [47]. A previous study with post MI rat model showed that GTPs reduced heart tissue remodeling injury, avoided ventricular hypertrophy, reduced infarct size, as well as significantly improved the left ventricular functions [14]. Using the same post MI model, the rate of intracellular free radicals produced in cardiomyocytes extracted from post MI rats with GTPs treatment for 3 days, 2 weeks, and 3 weeks all became slower in comparison with post MI cells without GTPs (Figure 2). With the increase of GTPs feeding period the rate of intracellular free radical production was also significantly reduced. In addition, GTPs treatment could help maintain the activity of SOD in cells located at the remote region of the heart in the post MI rats for the time periods from 3 days to 3 weeks, while in post MI group without GTPs treatment the SOD activity was found to be significantly decreased in cardiac tissues of rats suffered from post MI for 3 weeks (Figure 3). However, the measured SOD mRNA level in myocardial tissues was not significantly different in control rats, post MI rats with or without GTPs treatments. For measuring another anti-oxidant enzyme, heme oxygenase-1 (HO-1), the mRNA level was found significantly reduced in cardiac non-infarcted area (remote region) for post MI rats without GTPs treatment, while GTPs treatment prevented from the decrease of mRNA expression in myocardial tissues.

To further examine the events for the oxidative stress in myocardial cells, 4-hydroxynonenal (4HNE) post-translational modification on myocardial proteins were determined in the hearts of control rats, post MI rats with or without GTPs treatments. Results showed that 4-HNE modified proteins were increased in myocardial tissues for the post MI rats without GTPs treatment, but no significant difference with GTPs treatments, as compared to sham controls.

### 3.4. Ischemic preconditioning cardiac protection signaling pathways

Ischemic preconditioning (IPC) is one of the most effective cardio-protection in which short periods of IR in the heart confer resistance to a subsequent prolonged ischemic stress [36, 40, 48- 51]. Many mediators and effectors have been shown to be essential for IPC and include the ATP-dependent mitochondrial K+ channels (K_ATP_ channel), PKC, tyrosine protein kinases (TPK), and adenosine, bradykinin, adrenergic and muscarinic receptor, NO donors, and phosphodiesterase inhibitors, and endotoxins, cytokines, and ROS. IPC initiates a number of cardio-protective events depending on the intervening time period between the protective stimulus (IPC) and the index IR injury [36, 40, 50]. Acute IPC (min to hrs) is mediated by the posttranslational modification of proteins, while “second window” IPC (days) induces protection by de novo protein synthesis [51].

Cardiac protection involving a memory (preconditioning) might be attributed to trigger mitochondrial swelling that causes enhanced substrate oxidation and ROS production, leading to redox activation of PKC, which inhibits GSK-3β [52]. Alternatively, TPK or certain G-protein coupled receptor (GPCR)-dependent activation elicits cell protection by inhibiting GSK-3β, *via* Akt and mTOR pathways, PKC pathways, or PKA pathways [44]. The convergence of these pathways *via* inhibition of GSK-3β on the end effector to limit mPTP induction is the general mechanism of cardiomyocyte protection [52]. Recent reports also provided evidence for that the cardio-protection of GTPs against oxidative stress associated with myocardial ischemic injury is caused by reducing cytosolic Ca^2+^ overload and generation of ROS *via* the Akt/GSK-3β/β-catenine and caveolae signaling both *in vivo* myocardial ischemia injury [13, 14] and *In vitro* H_2_O_2_-induced oxidative stress models [29-30].

### 3.5. The GPCR-dependent signaling pathways for cardiac protection

The GPCR-dependent mechanism initiates a downstream signaling cascade involving TPK, PI3K/Akt, NOS, activation of K_ATP_ channel, generation of ROS, activation of PKC isoforms, GSK-3β, and MAPK, and inhibition of the opening of the mPTP [49-52]. Although these components are considered to play a role in cardiac protection, it still remains to be resolved as to how signaling networks interact spatially and temporally in producing such protection. In particular, little is known about the regions to which proteins translocate and the molecules with which they interact. In many cases, the signals from GPCRs to target proteins are mediated *via* lipid signals [53].

### 3.6. Caveolae /lipid rafts involved in cardiac protection

Membrane lipids forms organized and dynamic structures based on interactions between membrane lipids and proteins including caveoli with clear morphology, dynamic rafts of different sizes and specific annular lipid layers surrounding proteins due to mutual affinity of lipids and proteins [54, 55]. It is proposed that these rafts function as platforms for the attachment of proteins when membranes are moved around inside the cell and during signal transduction [54, 55]. It is generally accepted that the structural and functional properties of rafts require an intact microtubule and actin filament; both are the primary interacting partners of caveolae/lipid rafts [56, 57].

Many of the properties of rafts have been inferred from detergent-resistant membranes (DRMs) that occur in nonionic detergent (e.g. Triton X-100) lysates of animal cells [54-55]. Lipid rafts, enriched in cholesterol and sphingolipids, form one such microdomain along with a subset of lipid rafts, caveolae, enriched in the protein caveolin (Cav) [54, 55]. In the membrane raft model, the DRMs represent poorly solubilized rafts [55], and the composition of the DRMs has served as a guide to the structural and functional properties of rafts. These domains selectively and dynamically gather or exclude signaling proteins, and the activity and specificity of membrane proteins is regulated by interaction partners [54, 55]. The “Cav/lipid raft signaling hypothesis” postulates that the regulation of signal transduction events occurs as a result of interaction of signaling proteins with a “Cav scaffolding domain”, an interaction that is hypothesized to inhibit such pathways by sequestering components away from signal transduction partners [53-58].

**Fig. 4:**
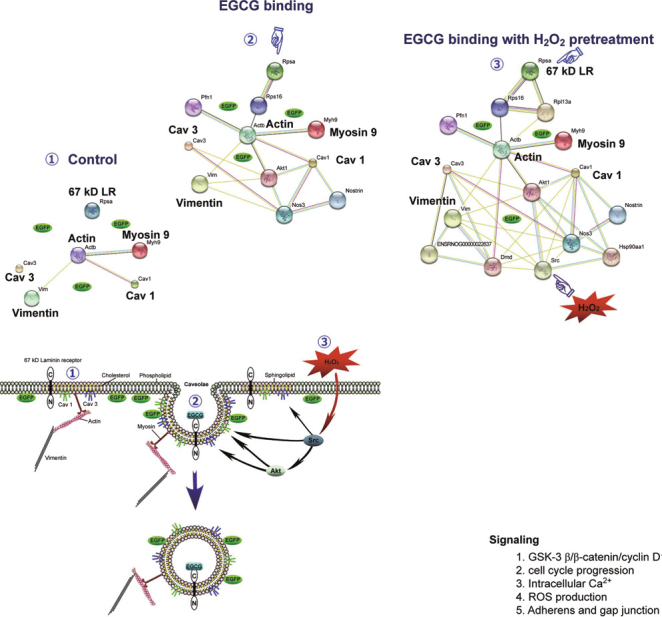
A hypothetical model for interaction networks obtained with identified proteins in EGFP-expressing H9c2 cells. The proteins identified were imported into the EMBL Search Tool for the Retrieval of Interacting Proteins (STRING) database, and an interaction map generated was used to construct the hypothetical mechanism for EGCG-induced fluorescence changes in EGFP-expressing H9c2 cells with or without H_2_O_2_ pretreatment [30].

A growing body of data indicates that multiple signal transduction events in the heart occur *via* plasma membrane receptors located in signaling microdomains [59-61]. In the heart, a key Cav is Cav-3, whose scaffolding domain is thought to serve as an anchor for other proteins [62, 63]. Immunoprecipitation with anticaveolin antibodies indicated that several GPCRs, and their cognate heterotrimeric G proteins and effectors, localize to lipid rafts/ caveolae in neonatal cardiac myocytes [64]. Using In vitro and *in vivo* models of IR injury, it has been shown that that the volatile anesthetic, isoflurane, modifies cardiac myocyte sarcolemmal membrane structure and composition and that activation of Src and phosphorylation of Cav-1 contribute to cardiac protection [62, 63]. Thus, multiple signal transduction events in the heart occur *via* plasma membrane receptors located in signaling microdomains [65]. A recent study using *In vitro* oxidative stress model in H9c2 cells has demonstrated that Cav/lipid rafts involve in GTPs-mediated Akt/GSK-3β signaling for cardio-protection during oxidative stress [30]. This study also proposed a hypothetical model with interaction networks based on the identified proteins in EGFP (enhanced green fluorescence protein) expressed cells (Figure 4). It is very likely that GTPs may act to protect cardiac cells from oxidative stress and ischemic injury through lipid rafts.

### 3.7. Convergence of signaling pathways for cardio-protection on GSK-3β

Numerous cardio-protective drugs are shown to converge on GSK-3β [44, 52, 66-69]. The phosphorylation and inhibition of GSK-3β lead to inhibition or delayed activation of mPTP, a key regulator of apoptosis [52]. A recent study using isolated perfused working rat hearts subjected to global IR demonstrated that addition of a selective inhibitor of GSK-3β prior to ischemia or at the onset of reperfusion improves recovery of left ventricle work by reducing proton production and attenuating the intracellular Ca^2+^ overload [66]. In addition, previous studies have suggested that IPC results in phosphorylation and inhibition of GSK-3β [44], and that drugs that inhibit GSK-3β are cardio-protective [44, 52]. β-catenin is a transcriptional activator that activates target genes in the nucleus [70, 71]. Growth factors promote β-catenin signaling by inhibiting its phosphorylation by GSK-3β, resulting in a reduction of its degradation by the proteasome and its subsequent activation in the nucleus [72, 73]. Cyclin D1 is one of target genes that might be activated by β-catenin for cell proliferation [69]. Consistently, inhibition on the β-catenin signaling pathway would lead to a decrease in cyclin D1 expression in cells that prevent cell cycle progression into S phase [68]. Another route for inhibition of GSK-3β on the β-catenin signaling pathway is to modulate the cell-cell adhesion and communication *via* adherens and gap junction proteins (i.e. Cx43) [13]. Evidence also suggested that GTPs pretreatment acts to protect heart from IR injury through PI3K/Akt survival pathway to limit GSK-3β activity in cardiac cells [13, 29, 30].

## 4. Molecular targeting for GTPs-mediated cardiac protections

### 4.1. Cardioproteomics application to discover signaling mechanisms involved in cardiovascular diseases

Proteomics is a new technology that allows the detection and the identification of several proteins at a given time in a sample [74- 76]. It combines several techniques, including 2-D gel electrophoresis, image analysis, and mass spectrometry. This technique has been extensively employed to identify proteins involved in cardiac regeneration in the infarcted myocardium [77], to analyze modifications in the plasma protein map during an acute coronary syndrome [78], to analyze the role of complement in myocardial IR and its effect on myocardial protein expression [79]. In an *in vivo* dog model of myocardial IR [80], 2-D gel electrophoresis was used to identify changes in the level of four metabolic enzymes and a contractile protein. In an *in vivo* rabbit model of cardiac IR, Schwertz *et al*. [81] found 10 protein spots that were differentially expressed: two as the protective proteins SOD and αB-crystallin More recently [82], a proteomic approach was also used to study of the effects of ramipril on post-infarction left ventricular remodeling in the rabbit. In an *In vitro* rat model [83], 8 protein spots with altered expression after cardiac ischemia or IR were found: 5 protein spots as the endoplasmic reticulum enzyme, one as 60 kDa heat shock protein and two as mitochondrial elongation factor Tu. In the mouse, a model of permanent ischemia and a model of IR were used to identify changes in cardiac protein expression after *in vivo* MI by 2-D gel electrophoresis combined with mass spectrometry [84]. Using the H9c2 cell model of H_2_O_2_-induced oxidative stress for a proteomics study, Chou *et al*. [85] showed that oxidative stress triggers tyrosine phosphorylation on target proteins associated with cell-cell junctions, the actin cytoskeleton, and cell adhesion in cardiac cells.

### 4.2. Cardioproteomics exploring GTPs-mediated anti-oxidative intervention in H9c2 cardiomyoblasts

**Fig. 5:**
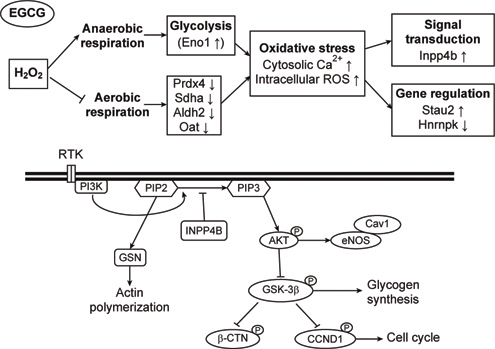
The putative mechanism for EGCG-conducted cardioprotection against H_2_O_2_-induced oxidative stress through the Akt/GSK-3β /caveolae pathways in cultured H9c2 cells. EGCG is hypothesized to protect cardiac cells from oxidative stress by PI3K/Akt survival pathway to attenuate the GSK-3β signaling on cardiac cell death.

To identify the potential proteins for the GTPs-mediated cardioprotection, cardiac proteomics study was performed in an H_2_O_2_- induced oxidative stress model of myocardial ischemia injury [29]. In this model, 8 proteins associated with metabolism, electron transfer, redox regulation, signal transduction, RNA binding and transcription regulation were identified to take part in EGCGameliorating H_2_O_2_-induced injury to H9c2 cells. H_2_O_2_ exposure increased oxidative stress evidenced by increases in ROS and cytosolic Ca^2+^ overload, increases in glycolytic protein, α-enolase (Eno 1), decreases in antioxidant protein, peroxiredoxin-4 (Prdx4), as well as decreases in mitochondrial proteins, including aldehyde dehydrogenase-2 (Aldh2), ornithine aminotransferase (Oat), and succinate dehydrogenase ubiquinone flavoprotein subunit (Sdha). All of these effects were reversed by EGCG pre-treatment. In addition, EGCG attenuated the H_2_O_2_-induced increases of Type II inositol 3,4-bisphosphate 4-phosphatase (Inpp4b) and relieved its subsequent inhibition of downstream signaling for Akt and GSK-3β/cyclin D1 in H9c2 cells. Pre-treatment with EGCG or GSK-3β inhibitor (SB 216763) significantly improved the H_2_O_2_-induced suppression on cell viability, phosphorylation of pAkt (S473) and pGSK-3β (S9), and level of cyclin D1 in cells. Finally, EGCG counteracted the H_2_O_2_-induced decreases in heterogeneous nuclear ribonucleoprotein K (HnrnpK), playing a role in cell cycle progression, but increases in double-stranded RNA-binding protein Staufen homolog 2 (Stau2), involving RNA functions. These findings suggest that GTPs might act to protect cardiac cells from oxidative stress through Akt survival pathway to inhibit the GSK-3β effect on cardiac cell death pathway (Figure 5).

### 4.3. Cardioproteomics identifying GTPs-mediated cardio-protection against myocardial ischemia stress in post LAD rats

It has been shown that LAD ligation for 3 days caused acute myocardial ischemia (AMI) and impairment of myocardial functions, while myocardial remodeling occurring in the rats after LAD ligation for 2 or 3 weeks [14]. Proteomic analysis has allowed to identify the molecular targets in the myocardium associated with disturbance by ischemia stress but protection by GTPs in post MI rats for 3 days (AMI) or 2-3 weeks (remodeling).

**Fig. 6:**
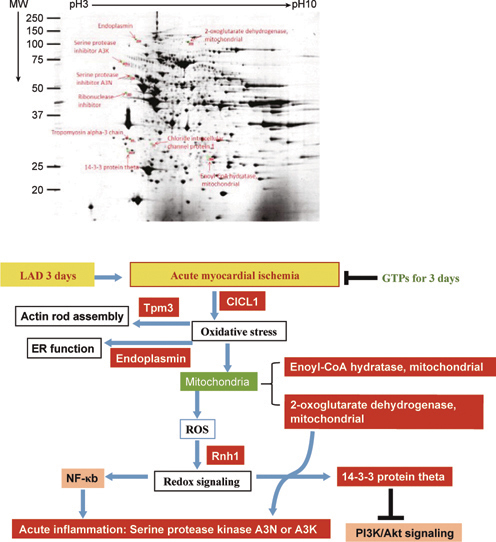
Proteomics analysis on molecular identification for targets involved in the GTPs-mediated cardio-protection against AMI in post LAD rats for 3 days.

In AMI model associated with post LAD ligation for 3 days, 10 proteins involved in the functions of myocardial ischemia stress (i.e. Chloride intracellular channel protein 1 (CICP1); Endoplasmin), cytoskeletal organization (i.e. Tropomyosin alpha-3 chain, tpm3), mitochondria metabolism (i.e. 2-oxoglutarate dehydrogenase, Enoyl-CoA hydratase), redox signaling (i.e. Ribonuclease inhibitor (Rnh1), 14-3-3 protein θ), and acute inflammation (i.e. Serine protease inhibitors A3N, A3K) were identified for the GTPs-mediated cardio-protection against AMI injury (Figure 6). The data suggested that the activation of NF-κb transcription factor and inhibition on PI3K/Akt signaling might account for the AMI-induced stress, and such redox signaling events could be prevented by GTPs (Figure 6).

**Fig. 7:**
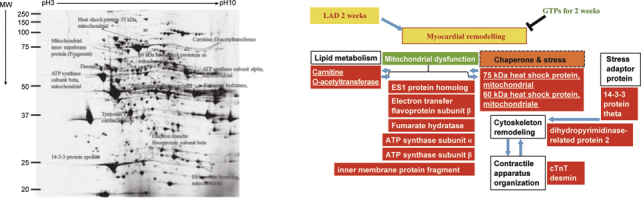
Proteomics analysis identifying molecular targets involved in the GTPs-mediated cardio-protection against myocardial remodeling in post LAD rats for 2 weeks.

**Fig. 8:**
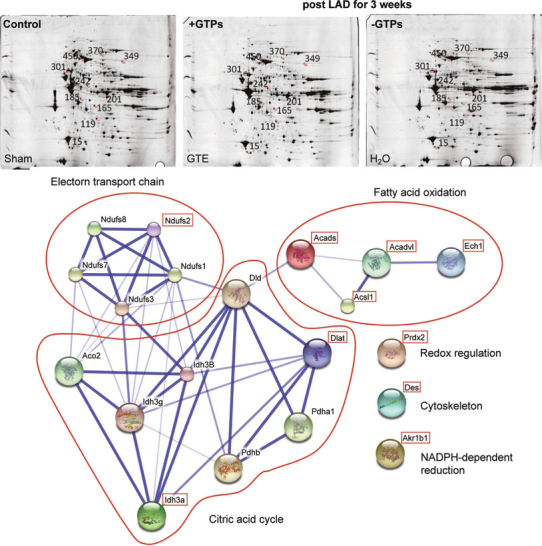
Molecular identification and hypothetical protein-protein interactions for proteins involved in myocardial remodeling of post MI for 3 weeks with or without GTPs in rats. Identified proteins are *Peroxiredoxin-2 (Prdx2) (Spot 15), Delta(3,5)-Delta(2,4)- dienoyl-CoA isomerase, mitochondrial, (Ech1) (Spot 119), Aldose reductase (Akr1b1) (Spot 165), Isocitrate dehydrogenase [NAD] subunit alpha, mitochondrial (Idh3a) (Spot 185), Short-chain specific acyl-CoA dehydrogenase, mitochondrial (Acads) (Spot 201), NADH dehydrogenase [ubiquinone] iron-sulfur protein 2, mitochondrial (Ndufs2) (Spot 242), Desmin (Des)(Spot 301), Very longchain specific acyl-CoA dehydrogenase, mitochondrial (Acadvl) (Spot 349), Long-chain-fatty-acid--CoA ligase 1 (Acsl1) (Spot 370), Dihydrolipoyllysine-residue acetyltransferase component of pyruvate dehydrogenase complex, mitochondrial (Dlat0 (Spot 450)*.

In remodeling model with post LAD ligation for 2 weeks, 14 proteins associated with chaperone proteins (i.e. Heat shock protein 75 kDa, mitochondrial; 60 kDa heat shock protein, mitochondrial), muscle proteins (i.e. cardiac troponin T, desmin), lipid metabolism (i.e. Carnitine O-acetyltransferase), mitochondria functions (i.e. ES1 protein homolog, Electron transfer flavoprotein subunit β, Fumarate hydratase, ATP synthase subunit α, ATP synthase subunit β, inner membrane protein fragment), developmental protein (i.e. dihydropyrimidinase-related protein 2), and stress related adaptor protein (i.e. 14-3-3 protein ε) were identified for the GTPs-mediated cardio-protection against the myocardial remodeling after ischemia stress (Figure 7). These data suggest that during myocardial ischemia remodeling cardiac cells disturbed by mitochondria dysfunction associated with alterations of lipid metabolism trigger chaperone/stress response *via* the adaptor protein (14-3-3 protein ε) resulting in cytoskeleton reorganization and contractile apparatus disruption. Such stress-induced redox signaling for myocardial ischemia remodeling could be improved by GTPs. Consistently, myocardial remodeling with post LAD for 3 weeks also identified 10 proteins associated with cytoskeletal function, energy metabolism (i.e. electron transport chain, citric acid cycle, and fatty acid oxidation), and redox regulation (Figure 8).

## 5. Perspectives

Green tea, being rich in polyphenols (GTPs), is a natural choice for its myocardial protection against ischemia or oxidative stress. Cardiac proteomics have allowed to reveal the underlying mechanisms for the actions of GTPs exerting their favorable cardioprotective effects. Currently, the important findings illustrating the potential end effectors involved in cardiac protection by GTPs include: (1) EGCG exerting cardio-protection against H_2_O_2_- induced oxidative stress through the Akt/GSK-3β/caveolae pathways in cardiac cells, (2) GTPs preventing their activation of NF-κb and their inhibition on PI3K/Akt signaling for the AMI stress, (3) GTPs ameliorating mitochondria dysfunction associated with alterations of lipid metabolism, chaperone-induced stress response, and the adaptor 14-3-3 ε protein signaling for cytoskeleton remodeling /contractile apparatus disruption during post MI remodeling. It appears promising to apply this natural product as a therapeutic approach to treat ischemic heart diseases in the near future.

## 6. Acknowledgements

This work was supported by the National Science Council of Taiwan government (to Y-M L., Grants: NSC 100-2320-B-005- 001, NSC 101-2320-B-005-001), and also supported by cooperative projects between the Taichung Veterans General Hospital and the NCHU (TCVGH-NCHU 103S0514).

## 7. Conflicts of interest statement

The authors declare that they have no conflicting interests.
